# Evolutionary degeneration of septins into pseudoGTPases: impacts on a hetero-oligomeric assembly interface

**DOI:** 10.3389/fcell.2023.1296657

**Published:** 2023-11-29

**Authors:** Alya Hussain, Vu T. Nguyen, Philip Reigan, Michael McMurray

**Affiliations:** ^1^ Program in Structural Biology and Biochemistry, Department of Cell and Developmental Biology, University of Colorado Anschutz Medical Campus, Aurora, CO, United States; ^2^ Department of Pharmaceutical Sciences, Skaggs School of Pharmacy and Pharmaceutical Sciences, University of Colorado Anschutz Medical Campus, Aurora, CO, United States

**Keywords:** septin, GTPase, PseudoGTPase, evolution, nucleotide, oligomerization

## Abstract

The septin family of eukaryotic proteins comprises distinct classes of sequence-related monomers that associate in a defined order into linear hetero-oligomers, which are capable of polymerizing into cytoskeletal filaments. Like actin and ⍺ and β tubulin, most septin monomers require binding of a nucleotide at a monomer-monomer interface (the septin “G” interface) for assembly into higher-order structures. Like ⍺ and β tubulin, where GTP is bound by both subunits but only the GTP at the ⍺–β interface is subject to hydrolysis, the capacity of certain septin monomers to hydrolyze their bound GTP has been lost during evolution. Thus, within septin hetero-oligomers and filaments, certain monomers remain permanently GTP-bound. Unlike tubulins, loss of septin GTPase activity–creating septin “pseudoGTPases”—occurred multiple times in independent evolutionary trajectories, accompanied in some cases by non-conservative substitutions in highly conserved residues in the nucleotide-binding pocket. Here, we used recent septin crystal structures, AlphaFold-generated models, phylogenetics and *in silico* nucleotide docking to investigate how in some organisms the septin G interface evolved to accommodate changes in nucleotide occupancy. Our analysis suggests that yeast septin monomers expressed only during meiosis and sporulation, when GTP is scarce, are evolving rapidly and might not bind GTP or GDP. Moreover, the G dimerization partners of these sporulation-specific septins appear to carry compensatory changes in residues that form contacts at the G interface to help retain stability despite the absence of bound GDP or GTP in the facing subunit. During septin evolution in nematodes, apparent loss of GTPase activity was also accompanied by changes in predicted G interface contacts. Overall, our observations support the conclusion that the primary function of nucleotide binding and hydrolysis by septins is to ensure formation of G interfaces that impose the proper subunit-subunit order within the hetero-oligomer.

## Introduction

Many nucleotide-binding proteins hydrolyze the γ phosphate of a nucleoside triphosphate (NTP) to either covalently attach the phosphate to another molecule (*i.e.*, kinases) or undergo a switch-like conformational change. For typical non-kinase NTPases, distinct binding partners recognize the NTP- or NDP (nucleoside diphosphate)-bound conformation, such as for Ras-family GTPases that toggle between functional states defined by binding to specific downstream effector proteins. For other NTPases, nucleotide hydrolysis controls higher-order homo- or hetero-oligomerization state. Tubulins provide an elaborate example: the “catalytic” residue that drives hydrolysis of the GTP bound by β tubulin is provided by ⍺ tubulin, such that hydrolysis only occurs in the context of an oligomer (Nogales et al., 1999). Dynamic instability of microtubules is a manifestation of distinct interactions between the tubulin proteins themselves that result from conformational changes occurring upon GTP hydrolysis and release of inorganic phosphate (Alushin et al., 2014; Manka and Moores, 2018). The building block of eukaryotic protofilaments and microtubules is a tubulin heterodimer, of which the ⍺ subunit is permanently GTP-bound (Nogales et al., 1998). The lack of GTPase activity stabilizes the ⍺–β tubulin heterodimer interface, ensuring that dynamics occur between, not within, heterodimers. The tubulin heterodimer evolved via gene duplication of an ancestral protein that presumably hydrolyzed GTP upon oligomerization (Findeisen et al., 2014). Gene duplication followed by loss of GTPase activity can thus direct the higher-order organization of hetero-oligomers.

While tubulin-family proteins constitute their own family of GTPases, loss of GTPase activity has occurred numerous times during evolution in the more canonical family of small GTPases, resulting in pseudoGTPases (Stiegler and Boggon, 2020). Candidate pseudoGTPases are recognized by non-conservative substitutions in key residues within otherwise conserved regions/motifs (Wittinghofer and Vetter, 2011), including the G1 motif (also called the “P-loop” due to proximity to phosphates of the nucleotide), the G2 motif (also called “Switch I” since the conformation of this loop is typically drastically different between the GTP- and GDP-bound states), the G3 motif (“Switch II”), and the G4 and G5 motifs, in which residues contact the nucleotide base and confer nucleotide specificity. For most pseudoGTPases, the functional consequences of loss of GTPase activity are unknown. One particularly informative pseudoGTPase evolved into an ATPase: non-conservative G4 and G5 substitutions in the *Bacillus subtilis* SpoIVA protein allow binding of ATP, hydrolysis of which drives homo-oligomerization during sporulation, when some ATP remains available in the cytosol but GTP is nearly absent (Ramamurthi and Losick, 2008; Updegrove et al., 2021). SpoIVA provides a compelling paradigm for remodeling of the GTP-binding pocket to accommodate a more available nucleotide.

**FIGURE 1:**
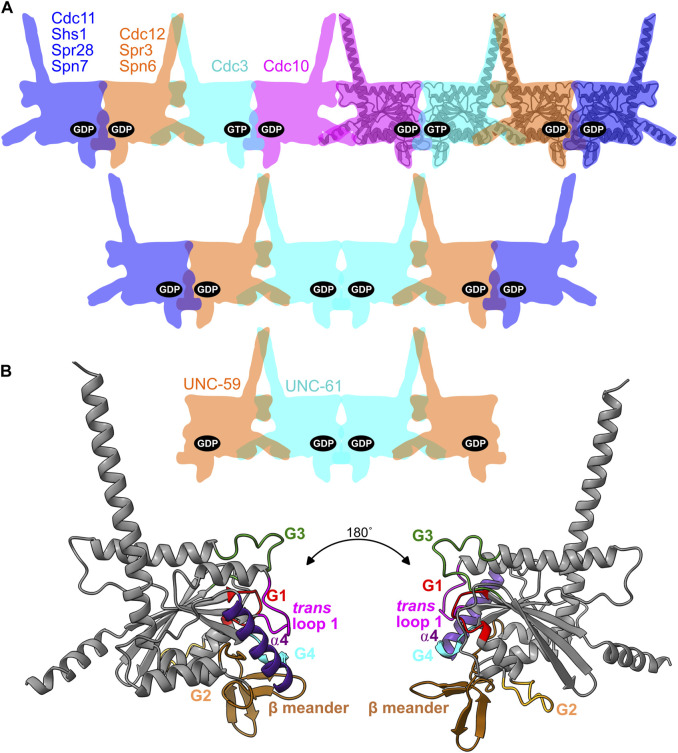
Septin binding to nucleotides in the context of oligomer organization and the G dimer interface. **(A)** Cartoon illustration of septin hetero-oligomers showing the nucleotides bound in the pockets for budding yeast hetero-octamers, fungal hetero-hexamers, and nematode hetero-tetramers. For reference, an AlphaFold predicted structure of Cdc10 is overlaid on four of the cartoon shapes. Named are the septins discussed in this study, with their locations and colors reflecting their positions within the complexes. **(B)** The AlphaFold structure of Cdc10, viewed from two angles and color-coded to highlight the regions making contacts across the G dimerization interface that encompasses the pocket.

We study the septin family of GTPases, which evolved from a canonical, Rossmann-fold GTPase of the TRAFAC class (Leipe et al., 2002) and polymerize into linear/rod-shaped homo- and hetero-oligomers to perform a wide variety of cellular functions (Mostowy and Cossart, 2012; Woods and Gladfelter, 2021). Unlike other cytoskeletal NTPases (tubulins, actins), within all known septin hetero-oligomers found in cells every septin nucleotide-binding pocket (the “pocket,” hereafter) is buried in a septin-septin interaction interface (called the G interface) (Sirajuddin et al., 2007) (Figure 1A), limiting nucleotide exchange. Septin hetero-octamers purified from the budding yeast *S. cerevisiae* contain stoichiometric quantities of guanine nucleotides (GTP or GDP) (Vrabioiu et al., 2004), suggesting that every septin pocket is occupied with bound nucleotide but some septins are GTPase-dead. Structural analysis of human septins identified a G2 Thr residue that is required for GTPase activity *in vitro* and is absent in the septins that fail to hydrolyze GTP *in vitro* and contain GTP in the pocket (Sirajuddin et al., 2009). We refer to this residue as the “catalytic Thr.” Here, we consider the evolutionary appearance of these and other kinds of septin “pseudoGTPases” and structural consequences for septin-septin interaction interfaces.

Extensive independent support for the importance of canonical GTPase motifs in *S. cerevisiae* septin assembly comes from temperature-sensitive mutants isolated in unbiased genetic screens, nearly all of which carry single amino acid substitutions in key G1, G3, G4, or G5 residues (Nagaraj et al., 2008; Weems et al., 2014). Genetic evidence suggests that the mutant yeast proteins are slow to fold, attract prolonged attention from cytosolic chaperones (Johnson et al., 2015; Denney et al., 2021), and at high temperature become “locked” in near-native conformations that are incompatible with co-assembly with wild-type septins into functional hetero-octamers (Weems et al., 2014; Johnson et al., 2020). A single G3 substitution in Cdc3 (D210G) is able to restore high-temperature function to a G4-mutant Cdc10 (D182N), presumably by stabilizing a compatible conformation of the G interface (Weems et al., 2014). Such “suppressor” mutants obtained in the lab suggest a mechanism by which a G dimer partner could “adapt” during evolution to a drastic change in a septin G interface caused by a substitution in a pocket or G interface residue.

We previously found evidence consistent with a model in which evolutionary variation in septin GTPase activity directs *de novo* higher-order septin oligomerization, in two distinct ways. 1) Slow septin GTP hydrolysis creates a transient GTP-bound monomer that, due to a specific G3 conformation, has a higher affinity for one partner septin over another that preferentially binds the GDP-bound state (*i.e.*, post-hydrolysis) (Weems and McMurray, 2017). 2) GTP but not GDP binding and lack of hydrolysis precludes the GDP-bound state and restricts the possible oligomers that can assemble, if G-interface-mediated dimerization between two GTP-bound septins is unfavorable (Johnson et al., 2020). During evolution of the yeast lineage that includes *S. cerevisiae*, loss of GTPase activity in the septin Cdc3 (due at least in part to substitution of the catalytic Thr) appears to be responsible for restricting septin assembly to hetero-octamers and preventing hetero-hexamer assembly (Johnson et al., 2020) (Figure 1A). Septin hetero-hexamers form when the septin in the Cdc3 position forms a G homodimer, rather than a G heterodimer with Cdc10 (Figure 1A). A GTP-bound pseudoGTPase in this position thus disfavors hexamer assembly (Figure 1A).

We previously noted evolutionary co-variation in Cdc3 between the catalytic Thr and residues proximal to the G interface, including an Arg in the ⍺4 helix that we proposed positions the *trans* loop 1 (Johnson et al., 2020). Like the switch loops, the *trans* loops 1 in human septins can adopt distinct configurations that depend on nucleotide phosphorylation state (Sirajuddin et al., 2009). In one configuration, a His within the *trans* loop 1 interacts with the β phosphate of GDP bound in the partner’s pocket. When γ phosphate is present on the nucleotide bound in the partner’s pocket, the His adopts a distinct conformation (Sirajuddin et al., 2009). While not making direct interseptin contacts and instead residing just “behind” the interface, the Arg presumably biases the *trans* loop 1 towards the β phosphate interaction and therefore fell out of favor when, during fungal evolution, the catalytic Thr was lost and the γ phosphate became a permanent resident of the pocket.

We also previously noted co-variation in Cdc10, the G heterodimer partner of Cdc3, and speculated that the changes represent a form of adaptation to the changes in the G interface imposed by loss of Cdc3 GTPase activity (Johnson et al., 2020). In yeasts with a GTPase-dead Cdc3 homolog, instead of the highly-conserved *trans* loop 1 His, the Cdc10 homolog instead has Lys, which we proposed favors interaction between Cdc10•GDP and Cdc3•GTP (Johnson et al., 2020). Satisfyingly, recent crystal structures of the *S. cerevisiae* Cdc3–Cdc10 G heterodimer show that Cdc10 Lys155 directly contacts the γ phosphate of GTP in the Cdc3 pocket (Marques da Silva et al., 2023). Reciprocally, the *trans* loop 1 His in Cdc3 contacts the β phosphate of GDP in Cdc10’s pocket (Marques da Silva et al., 2023). These examples illustrate how changes in individual amino acids correlate with distinct nucleotides bound within the septin G dimer interface. Comparisons of the recent Cdc3–Cdc10 structures to heterodimers involving human pseudoGTPases revealed another example (coordinating a G3 Gly with γ phosphate using a water molecule versus a G1 Asp sidechain) of how the pocket of the pseudoGTPase changed in distinct ways in parallel evolutionary trajectories to accommodate γ phosphate as a permanent resident (Marques da Silva et al., 2023). Here we explore other examples of deviation within the canonical components of the septin G interface and discuss how they may relate to the evolution of septin pseudoGTPases.

## Materials and methods

### Structure prediction

Models for wild-type septin monomers (Cdc10, Spn7, Spr28, Cdc11, and Shs1) were downloaded from the AlphaFold protein structure database (entries P25342, O60165, Q04921, P32458, and Q07657). Structures of the mutant septins Cdc10(D182N) and Cdc12(G247E) and dimer structures were generated from protein sequences using AlphaFold 2.2.2 run on the High-Performance Computing cluster of the University of Colorado Anschutz Medical Campus - Structural Biology Shared Resource—CryoEM Facility, RRID:SCR_021999 (Mirdita et al., 2022). Dimer structures were generated using a maximum template date of 2020-05-14, or 1900-01-01 to omit structural data from predictions. An exception was the Cdc11–Cdc12 structure, which was generated using AlphaFold2 Multimer v2 via Colabfold v1.3.0 (Mirdita et al., 2022).

### *In silico* nucleotide docking

Schrödinger Suite Release 2022-4 was used for all ligand and protein preparation, and all subsequent nucleotide docking calculations. The Glide Module was used for the docking (Friesner et al., 2004; Halgren et al., 2004; Friesner et al., 2006). Nucleosides/nucleotides ligands (consisting of guanosine, GMP, GDP, GTP, inosine, IMP, IDP, ITP, xanthosine, XMP, XDP, XTP, ATP, and CTP) were prepared at physiological pH using the LigPrep module. Septin structures (as.pdb files consisting of Cdc3, Cdc10(D182N), Cdc12(G247E), Spn7, Spr28, Cdc11, and Shs1) were prepared using the models described above and energetically relaxed to a potential minimum with OPLS4 force fields and the VSGB solvation model (Jorgensen et al., 1996). All septin structures were scanned and mapped to confirm their binding pocket and computational grids were created around those binding pockets for the ligand docking. All the aforementioned ligands were docked into all above septin structures within the created grids, in which top ranked conformations and docking scores of different ligand-protein complexes were generated.

### Identification and prediction of interface contacts

The Structure Analysis Tool “H-Bonds” in Chimera X v.1.6.1 (Pettersen et al., 2004) was used to identify interface contacts. H-bonds parameters were relaxed by a distance tolerance of 1 Å and an angle tolerance of 20°.

### Sequence alignments and phylogenetic trees

Multiple sequence alignments were created either manually or using the BLASTP and/or COBALT (Papadopoulos and Agarwala, 2007) servers at NCBI. Phylogenetic trees were created using the COBALT server.

## Results and discussion

### Three classes of septin pseudoGTPases

We consider for septins three classes of pseudoGTPases, partially overlapping with the three classes that have been previously considered for pseudoGTPases in general (Stiegler and Boggon, 2020).

**FIGURE 2:**
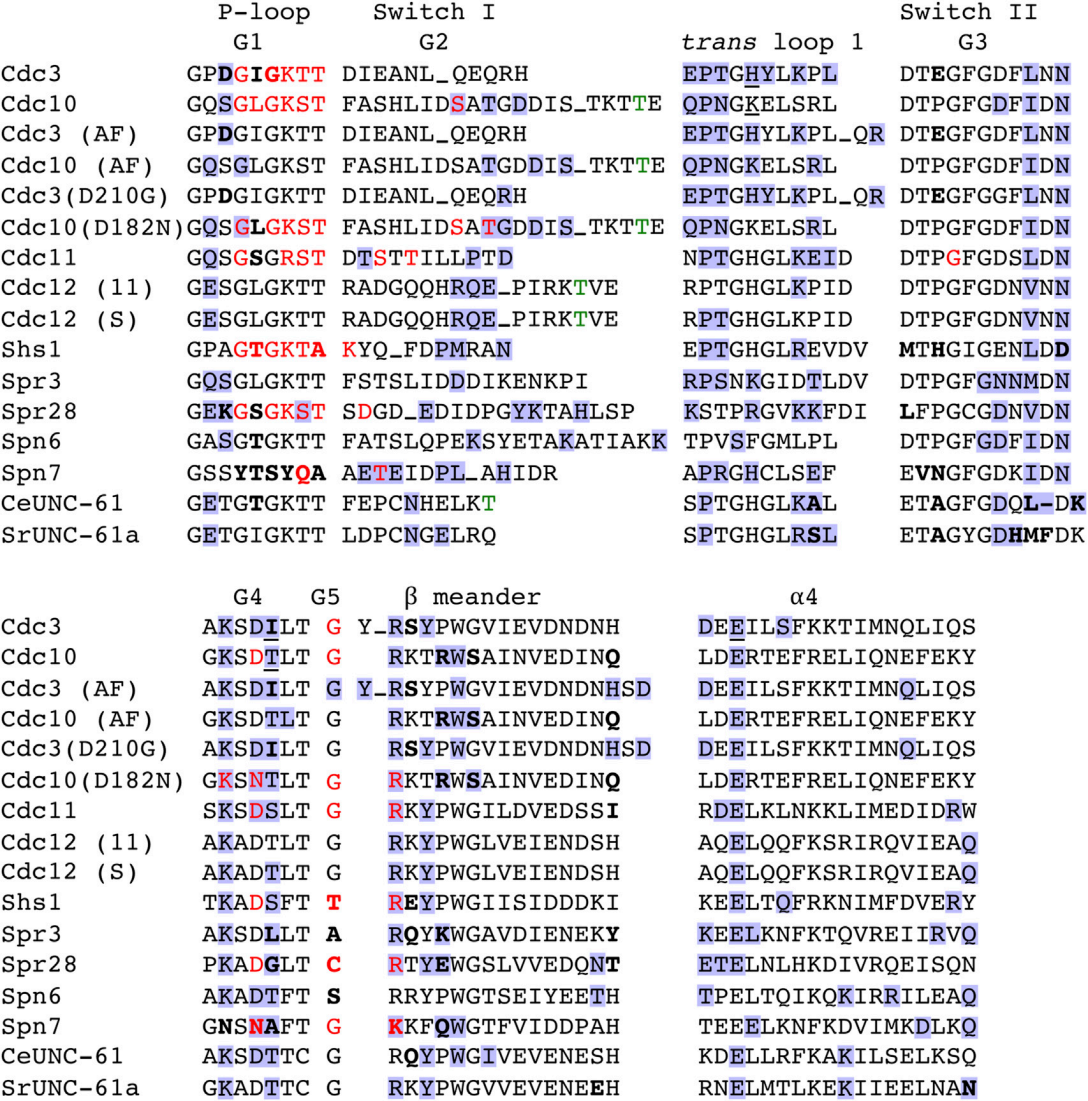
Non-conservative substitutions in canonical GTPase residues and predicted nucleotide and interface contact in yeast and nematode septins. Amino acid sequence alignment for selected regions of the G interface. “AF,” AlphaFold predicted structure. “(11),” sequence for Cdc12 showing predicted contacts with Cdc11; “(S),” sequence for Cdc12 showing predicted contacts with Shs1. Bold, notable deviation from consensus. Red, experimental or predicted direct contact with GTP or GDP bound in the pocket. Blue background, experimental or predicted contact across the G dimer interface. Green, known or predicted catalytic Thr. Underline, directly contacts the nucleotide in the G dimer partner’s pocket. Underscore, multiple residues not shown. Dash, gap where protein has no counterpart residue.

Class I septin pseudoGTPases have accumulated numerous substitutions in key pocket residues that are incompatible with binding to GTP or GDP. We propose Spn7 from the fission yeast *Schizosaccharomyces pombe* as the founding member of this class. Spn7 is expressed only during sporulation and occupies the subunit position at the ends of septin hetero-octamers, associating via its G interface with Spn6 (Onishi et al., 2010). Others have noted that Spn7 has non-conservative substitutions in G1, G3, and G4 pocket residues (Onishi et al., 2010) (Figure 2). We also tentatively placed Spr28, the *S. cerevisiae* homolog of Spn7, in this class, though its pocket motifs are more canonical (Figure 2). Class I septin pseudoGTPases may bind no nucleotide (*i.e.*, their pockets are empty), or a different small molecule that is more available in the cellular conditions in which the septin is expressed. The ATP-binding *B. subtilis* SpoIVA represents a non-septin example of this pseudoGTPase class. SpoIVA hydrolyzes ATP (Updegrove et al., 2021); the lack of experimental data for catalytic activity by most septins precludes us from dividing this group into two classes based on their ability to hydrolyze a non-GTP nucleotide.

Class II septin pseudoGTPases have mostly normal pockets but lack the catalytic Thr and therefore cannot hydrolyze GTP. They retain the ability to bind GTP and may also bind GDP, but cannot convert bound GTP to GDP. Human Septin-6 and its closest human relatives (Septin-8, -10, −11, and −14, all of which lack the catalytic Thr) fit in class II. The Momany group 1a includes these human septins as well as *S. cerevisiae* Cdc3 and its homologs in several other yeast species (Shuman and Momany, 2021). Group 1a also includes UNC-61, one of two septins in the nematode *Caenorhabditis elegans*, yet UNC-61 retains the catalytic Thr (Figure 2). Hetero-tetrameric complexes of UNC-61 with the other *C. elegans* septin (UNC-59) purified following co-expression in *E. coli* or insect cells contained some GDP but no GTP (John et al., 2007), consistent with GTP hydrolysis by both septins. However, a group 1a member from another helminth species, SmSeptin-10 from the flatworm *Schistosoma mansoni*, lacks the catalytic Thr and cannot hydrolyze GTP, yet readily binds GTP or GDP with similar affinity (Zeraik et al., 2014). The presence of a putatively active helminth GTPase (*Ce*UNC-61) in a phylogenetic group with a helminth pseudoGTPase (*Sm*Septin-10) and multiple human pseudoGTPases points to independent events in which GTPase activity was lost: once in the lineage giving rise to humans and at least once in the lineage giving rise to schistosomes. Finally, Spr3, the sporulation-specific *S. cerevisiae* homolog of *S. pombe* Spn6, is in the fungus-specific Momany group 4 with Cdc12 (Shuman and Momany, 2021), but unlike Cdc12 it lacks the catalytic Thr (Figure 2). Loss of the catalytic Thr must have occurred via an evolutionary event that was distinct from the loss events that led to Cdc3, human Septin-6, and *Sm*Septin-10. Below we examine these and other class II septin pseudoGTPases in more detail.

Class III septin pseudoGTPases have altered the pocket in ways that retain the ability to bind GDP but prevent GTP binding. Substitutions directly impinge on the ability to accommodate the γ phosphate. Class III septins presumably bind GDP immediately upon folding. We initially placed *S. cerevisiae* Cdc11 and its paralog Shs1 in this class, for the following reasons. Both proteins are found in vegetatively/mitotically proliferating cells, but Cdc11 protein is also present during sporulation, when GTP is scarce (Varma et al., 1985). Cdc11 and Shs1 are closely related to the sporulation-specific *S. cerevisiae* septin Spr28 [all are in fungus-specific Momany group 3 (Shuman and Momany, 2021)] and both have non-conservative substitutions in key pocket residues (Figure 2). In the crystal structure of truncated, nucleotide-free Cdc11, a non-canonical G1 Arg was positioned in a way that would clash with the presence of a phosphate (Brausemann et al., 2016). Based on these and other *in vitro* binding data, it has even proposed that Cdc11 does not bind any nucleotide (Baur et al., 2019).

However, septin hetero-octamers containing Cdc11 and Shs1 purified from vegetatively proliferating cells contain guanine nucleotides at a 1:1 protein:nucleotide ratio, with a GDP:GTP ratio of 2.2:1 (Vrabioiu et al., 2004). Assuming all Cdc3 is GTP-bound, the other septins must be bound mostly to GDP, with a few molecules of at least one other septin having GTP in their pockets. We proposed that Cdc12 is a slow, monomeric GTPase and Cdc11 specifically binds the transient GTP-bound form of Cdc12 prior to GTP hydrolysis, whereas Shs1 binds Cdc12•GDP (Weems and McMurray, 2017). If some Cdc12 retains GTP following hetero-octamer assembly, this could explain the 2.2:1 ratio. Hence, we initially suspected that within hetero-octamers, both Cdc11 and Shs1 are always GDP-bound. Below we explore Cdc11 and Shs1 as candidate representatives of class III septin pseudoGTPases.

### *In silico* prediction of nucleotide binding by divergent and mutant septins

Given the SpoIVA precedent and the known drop in guanine nucleotide levels during yeast sporulation, we used *in silico* docking to predict binding of non-GTP nucleotides to Spn7, Spr28, Cdc11, Shs1 and two mutant versions of active septin GTPases. Apart from a crystal structure of a truncated, nucleotide-free form of Cdc11 (Brausemann et al., 2016), experimental structures are not available for these septins. We relied on AlphaFold-driven sequence-directed structure prediction, which generates nucleotide-free structures. We first asked to what extent the conformation of the pocket depends on occupancy with bound nucleotide. Does an empty pocket look the same as a full one?

We removed the nucleotides from the Cdc3–Cdc10 heterodimer crystal structure *in silico* and performed free energy minimization, then superimposed the resulting monomer structures with the originals. Since Cdc3 pocket residues and the β and γ phosphates of bound GTP coordinate a magnesium ion (Marques da Silva et al., 2023), we also examined the effects of removing the magnesium ion. For Cdc3, all key pocket residues were positioned similarly (Supplementary Figure S1A and not shown). By contrast, for Cdc10 there were more differences, particularly in part of the septin unique element (SUE) (Figures 1B, 2), which distinguishes septins from other Ras-family GTPases (Versele et al., 2004). Within the SUE, a three-stranded β meander represents a key component of the G interface, involving intercalating hairpins centered on an absolutely conserved Trp residue (Figure 1B) (Shuman and Momany, 2021). Cdc10 Trp255 was displaced by nearly 7 Å (Supplementary Figure S1B), and nearby Arg251 moved deep into the pocket, in a way that would clash with bound nucleotide (Supplementary Figure S1B). Arg is highly conserved in this position (Pan et al., 2007; Shuman and Momany, 2021) (Figure 2) and contacts both the guanine base of bound nucleotide and, via a salt bridge, Cdc3 Glu295 across the G interface (Supplementary Figure S1C).

All the AlphaFold predictions of septin monomers we examined had the Arg251 equivalent residue (Lys in Spn7) in this same “clashing” position (Supplementary Figure S1D), which presumably reflects both lack of nucleotide and lack of G dimer partner during folding prediction. Others have reported the same effect with human septins, involving the equivalent Arg, when nucleotide was removed *in silico* from experimental structures (Grupp et al., 2023). When we attempted to dock nucleotides into these structures, none of the top-ranked poses featured nucleotide bound in the pocket in the canonical way (not shown). By contrast, when we manually reoriented the Arg251 equivalent residues to the dimerized/nucleotide-bound configuration adopted by Cdc10 in the Cdc3–Cdc10 crystal structure, top-ranked poses featured nucleotide bound in mostly canonical ways (see below). Another caveat of this approach is that in the AlphaFold predictions no nucleotide is present to guide the conformations of the switch regions (G2 and G3) that, by definition, can adopt distinctly different conformations depending on the presence or absence of the γ phosphate. Thus, G2 and G3 residues that normally contact the γ phosphate may be misoriented in our predicted structures, unless there are intramolecular contacts within the protein that position them regardless of what nucleotide occupies the pocket.

In addition to Spn7, Spr28, Cdc11 and Shs1, we tested two temperature-sensitive mutant *S. cerevisiae* septins, Cdc10(D182N) and Cdc12(G247E), isolated in unbiased genetic screens (Hartwell, 1971; McMurray et al., 2011; Weems et al., 2014). The same Asp-to-Asn G4 substitution found in Cdc10(D182N) was originally rationally engineered in the *E. coli* GTPase EF-Tu, where it switched specificity from guanosine to xanthosine nucleotides (Hwang and Miller, 1987). The mutation was also found in Septin-12 in an infertile man, where the mutant septin was unable to bind GTP *in vitro* and his sperm consequently lacked the septin-based filamentous ring called the annulus (Kuo et al., 2012). [Notably, another man was identified in the same study as carrying a Septin-12 mutation in the catalytic Thr, which eliminated GTP hydrolysis *in vitro* (Kuo et al., 2012)]. Thus, we expected Cdc10(D182N) to bind XTP but not GTP. Based on earlier docking using homology models, we previously speculated that the G5 mutation in Cdc12(G247E) switches nucleotide binding to CTP (Weems et al., 2014).

We tested 14 nucleosides/nucleotides. Inosine nucleotides were of particular interest since inosine resembles guanosine (Supplementary Figure S1E) and accumulates during yeast sporulation (Walther et al., 2014). Following exposure to phosphatase *in vitro*, purified human Septin-7 was found bound to unphosphorylated guanosine in the context of a dimer with GTP-bound Septin-7 (Zent and Wittinghofer, 2014). Thus, even a wild-type septin GTPase can bind an unphosphorylated nucleoside, albeit in unnatural conditions. Accordingly, we included the unphosphorylated nucleosides guanosine, inosine, and xanthosine in addition to their mono-, di-, and triphosphate nucleotide derivatives, and ATP and CTP.

**FIGURE 3:**
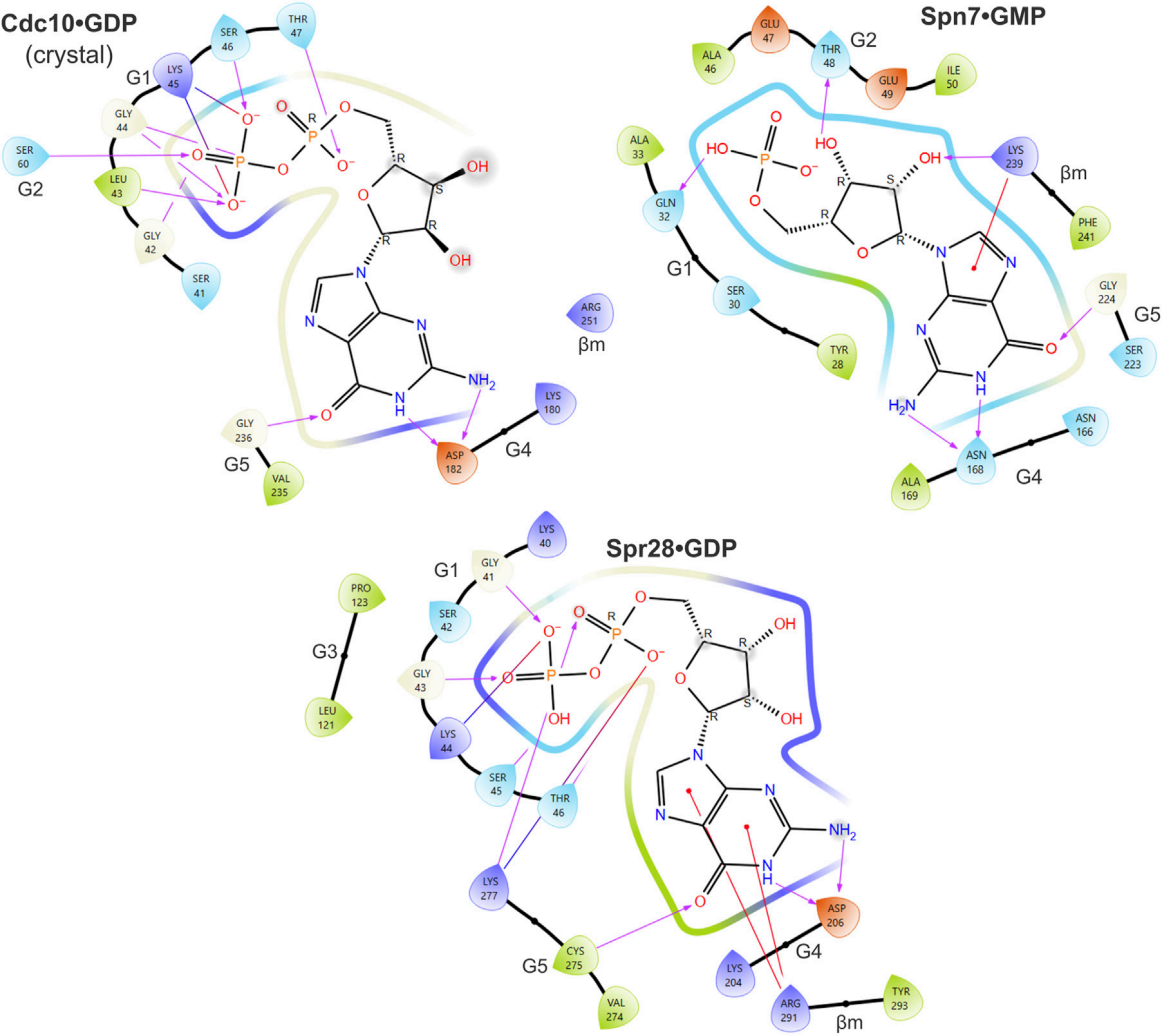
Predicted contacts between sporulation septins and bound nucleotide according to *in silico* docking. Ligand interaction diagrams showing residues from the indicated septin proteins that are predicted to be within 4 Å of the indicated nucleotide. Color coding of amino acids: dark blue, positive charge; red, negative charge; green, hydrophobic; light blue, polar. Purple arrows show hydrogen bonds. Red lines indicate pi-cation interactions. Gray spheres indicate solvent exposure. Also labeled are the regions of the septin protein to which the residues belong (e.g., “G1”). “βm,” β meander.

Table 1 lists the top three nucleotides bound, according to the “docking score” generated by the docking software, where the lower the score, the better the binding. Also shown is the rank and score for GTP and GDP. For each septin, if no pose was within the ∼15 top-ranked poses, no score was assigned. Unphosphorylated or monophosphorylated nucleotides bound best to Spn7, consistent with the non-conservative G1 substitutions in the residues that normally contact the β phosphate (Figures 2, 3). Even the best binding to Spn7 had a worse docking score than any of the other top three nucleotides for any other septin, including the two mutants (Table 1). Neither GTP nor GDP generated a scored pose bound to Spn7.

**TABLE 1:** In silico docking scores for nucleotide binding to AlphaFold models of septin monomers. When no pose featuring the indicated nucleotide was within the top ∼15 poses, no score is shown (“–”).

Septin	#1 nucleotide bound (docking score)	#2 nucleotide bound (score)	#3 nucleotide bound (score)	GTP rank (score)	GDP rank (score)
Spn7	GMP (−6.240)	XMP (−6.113)	Guanosine (−5.446)	—	—
Spr28	XTP (−9.903)	GDP (−9.717)	IDP (−9.715)	6 (−8.592)	2 (−9.717)
Cdc11	GTP (−10.712)	IDP (−9.668)	ITP (−9.373)	1 (−10.712)	4 (−9.066)
Shs1	GDP (−9.244)	IDP (−8.282)	GMP (−7.139)	—	1 (−9.244)
Cdc10(D182N)	IDP (−10.432)	GDP (−9.541)	XDP (−9.476)	—	2 (−9.541)
Cdc12(G247E)	GMP (−8.804)	XDP (−8.036)	Guanosine (−7.519)	—	5 (−7.040)

Spr28 retains a mostly canonical G1 and in our predictions numerous G1 residues contact the phosphates (Figure 3). Cdc3 uses an unusual G1 Asp to coordinate the γ phosphate (Marques da Silva et al., 2023), and Spr28 is highly unusual in having a Lys in this position (Figure 2). A long, charged G3 side chain (Asp or Glu) that canonically contacts the γ phosphate via a coordinating magnesium ion (Marques da Silva et al., 2023) is also replaced in Spr28 by Leu (Figure 2). These substitutions may explain why GTP binding was relatively disfavored compared to GDP (Table 1).

**FIGURE 4:**
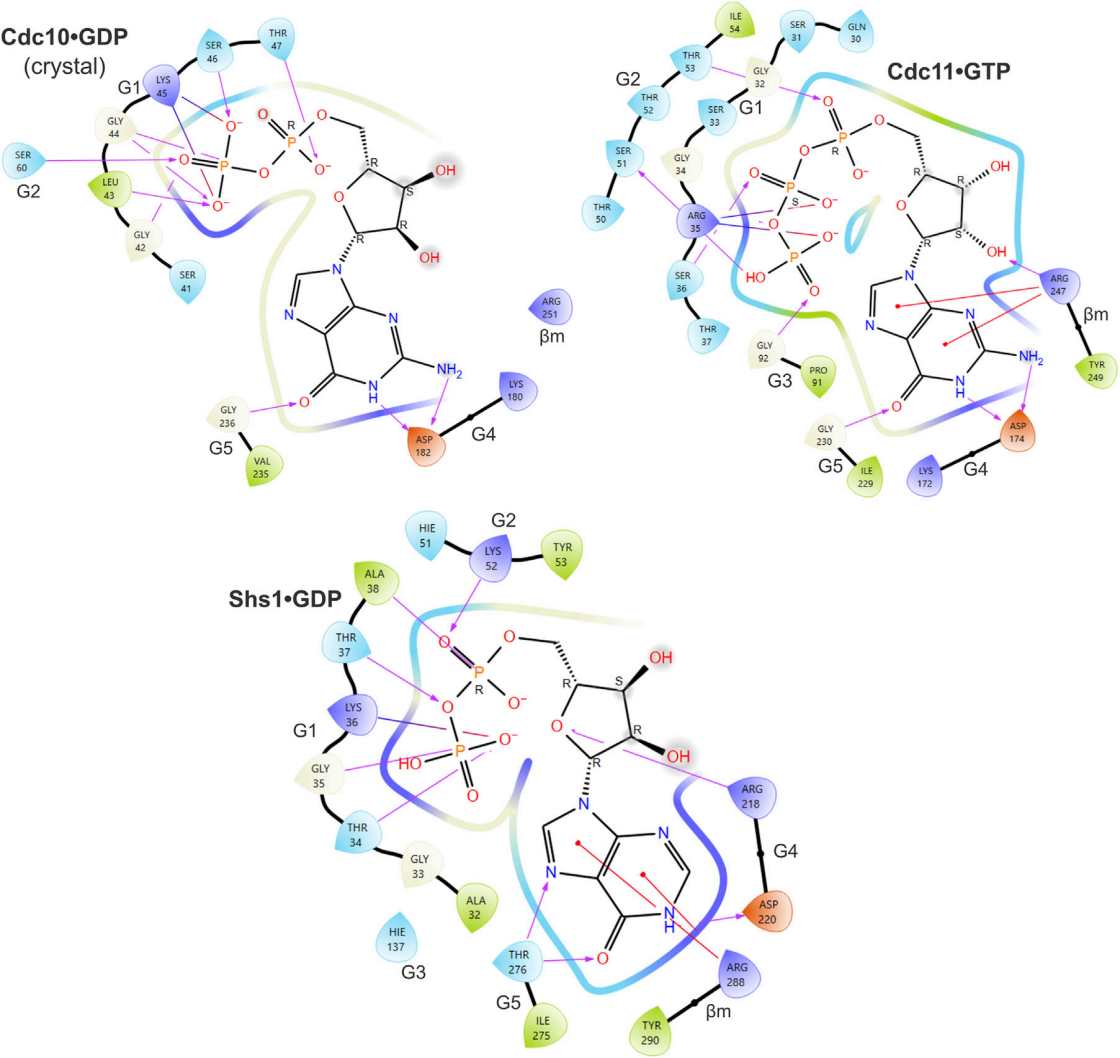
Predicted contacts between Cdc11 or Shs1 and bound guanine nucleotide according to *in silico* docking. As in Figure 3, and also showing the ligand interaction diagram for Cdc10 based on the Cdc3–Cdc10 crystal structure, but for Cdc11•GTP and Shs1•GDP.

Since IDP, XDP and XTP are not typically found in cells, even those undergoing sporulation, predicted binding to these nucleotides is likely not meaningful except to indicate that binding to a more physiological alternative (*e.g.*, GTP) was less favored. Our findings suggest that despite numerous changes to canonical motifs the pockets of Spr28 and Shs1 retain the ability to bind GDP. Shs1 is unlikely to stably bind GTP. By contrast, in our predictions, GTP made contacts with all five canonical GTPase motifs in Cdc11, resulting in the best docking score of all septin-nucleotide combinations (Figure 4). The G1 Arg that was previously proposed to preclude the presence of γ phosphate in the pocket instead contacted both the γ and β phosphates (Figure 4). Three G2 Thr residues were near the phosphates (Figure 4). If 40% of Cdc11 molecules are GTP-bound *in vivo*, this could explain the 2.2:1 GDP:GTP ratio [assuming no Cdc12•GTP and a Cdc11:Shs1 ratio of 1.6:1 (Weems and McMurray, 2017)].

The Cdc3–Cdc10 crystal structure (Marques da Silva et al., 2023) and the 1:1 nucleotide:protein ratio in purified yeast octamers (Vrabioiu et al., 2004) demonstrate that Cdc3 and Cdc11 both stably bind nucleotide in the context of stable septin hetero-oligomers. Why do individually purified Cdc3 and Cdc11 fail to bind any nucleotide *in vitro* (Versele and Thorner, 2004; Baur et al., 2019)? We previously showed that individually purified Cdc3 misfolds and forms a non-native homodimer via C-terminal coiled-coil-forming sequences (Hassell et al., 2022), highly reminiscent of the homodimers made by purified full-length Cdc11 (Brausemann et al., 2016). Rather than buried in a G interface, the pockets in these proteins are mostly exposed to solvent, potentially favoring loss of any nucleotide that was bound during *de novo* folding *in vivo.* In this scenario, the Arg residue equivalent to Cdc10 Arg251 may reorient into the position we and others saw upon energy minimization *in silico*, thereby inhibiting subsequent binding of exogenously added nucleotide. In the context of *de novo* folding, the nascent pocket is likely kept “open” and competent to bind nucleotide by virtue of G interface interactions with cytosolic chaperones. This model is consistent with our published findings that some isolated yeast septins require the presence of chaperones to maintain native conformations, and chaperones directly contact the septin G interface (Hassell et al., 2022).

The substitutions in the mutant GTPases also affected nucleotide binding, though not exactly in the ways we expected. The G4 Asp-to-Asn mutation in Cdc10(D182N) and the presence of a charged, bulky residue instead of Gly in the G5 of Cdc12(G247E) both reoriented the nucleotide in the pocket: rather than the canonical interactions with the base, the G4 contacted the ribose (Supplementary Figure S2). For Cdc12(G247E), G1 residues that canonically contact the β phosphate instead contacted the ⍺ phosphate (Supplementary Figure S2). For neither mutant was GTP or CTP able to bind in a top-ranked pose (Table 1).

Above we described why the presence (albeit transient) of Cdc12•GTP is important for yeast septin function. The conformation of the septin dimerization interface by which Cdc10 forms the central homodimer in yeast hetero-octamers (the “NC” interface (Sirajuddin et al., 2007), see Figure 1A) has been proposed to change allosterically depending on whether GTP or GDP is bound in the pocket (Sirajuddin et al., 2009; Kim et al., 2012). Thus while the Cdc10 molecules ultimately found in yeast hetero-octamers are GDP-bound, even the fleeting existence of Cdc10•GTP may be important for early steps in assembly (Weems and McMurray, 2017). It therefore makes sense that a defect in GTP binding by Cdc10(D182N) or Cdc12(G247E) results in defects in septin function. Together, these observations support the ability of our *in silico* approach to determine which pocket substitutions affect septin binding to specific nucleotides.

### Evolutionary co-variation of G interface contacts in septin pseudoGTPases

**FIGURE 5:**
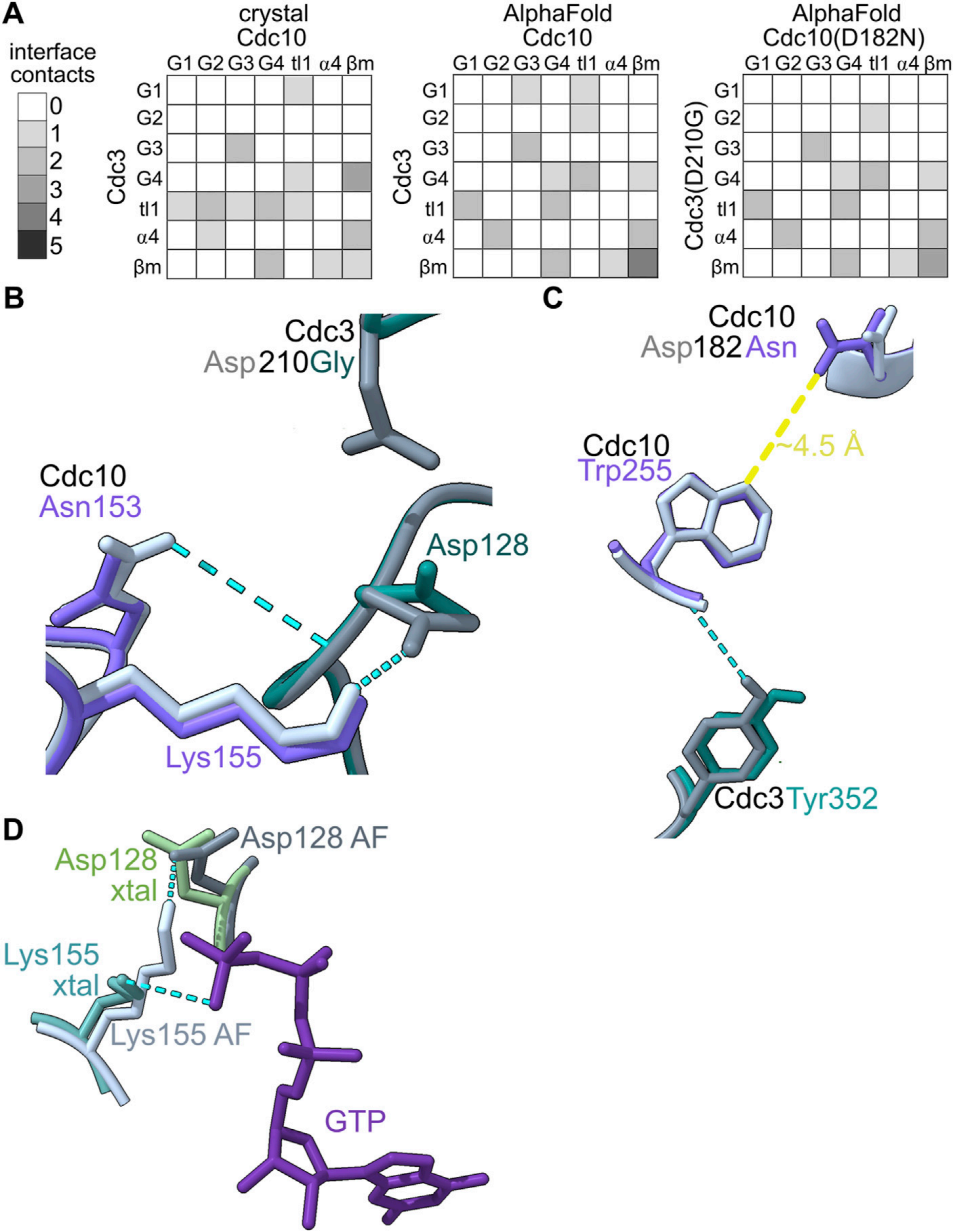
*In silico* predictions identify subtle changes in septin heterodimer contacts. **(A)** The number and origin (in terms of canonical septin G interface region/motif) of hydrogen bonds between residues in Cdc3 and Cdc10 are plotted as heatmaps. “tl1,″ *trans* loop 1. “ β m," β meander. **(B–C)** Superposition of AlphaFold predictions of Cdc3–Cdc10 and Cdc3(D210G)–Cdc10(D182N) showing residues making differential interface contacts as well as the mutant residues. Hydrogen bonds are shown as cyan dashed lines. **(D)** Superposition of Cdc10 Lys155, Cdc3 Asp128 and GTP from the crystal structure (PDB 8SGD, “xtal”) with the nucleotide-free AlphaFold prediction (“AF”). Yellow line shows approximate distance.

We sought to compare the ways that the G dimer partners of the various septin pseudoGTPases changed (if at all) to accommodate changes in the nucleotide bound in the pocket. We used AlphaFold Multimer to predict G dimer structures. We first tested the extent to which an AlphaFold model, which lacks bound nucleotide, recapitulates an experimental dimer interface in which nucleotides are bound by both septins: the crystal structure of the Cdc3–Cdc10 GTPase domains (PDB 8SGD). In Figure 5, we provide visual representations of the number and arrangement of interface contacts in the form of heat maps.

We saw only subtle differences between the experimental Cdc3–Cdc10 model and the AlphaFold prediction: all the same interface regions were involved in both interfaces, and if a specific contact in one region was lost, new contacts in adjacent residues usually appeared (Figure 5A). In the crystal structure and in every AlphaFold prediction, interface contacts almost exclusively involved residues in the G1, G2, G3, G4, ⍺4 helix, or β meander (Figure 5A). We conclude that the absence of bound nucleotide does not grossly distort the interface contacts in AlphaFold predictions versus experimental models of septin G interfaces.

As a test of the sensitivity of our approach in detecting changes in G interface contacts upon non-conservative substitutions in pocket residues, we next predicted the interface between Cdc10(D182N) and Cdc3(D210G), in which the G3 mutation in Cdc3 restores thermostability to the interface despite the G4 mutation in Cdc10 (Weems et al., 2014). Only three contacts changed (Figures 5A–C). Two of these involve Cdc3 Asp128 (Figure 5B), the same unusual G1 Asp that contacts the γ phosphate in the Cdc3–Cdc10 crystal structure. In the AlphaFold prediction, the backbone of Asp128 is near the side chain of Asp210 (Figure 5B), which is mutated to Gly in Cdc3(D210G). Repositioning of Asp128 when the Asp210 side chain is missing provides an explanation for the changes in these two G interface contacts.

The other change involved the β meander (Figure 5C). The β meander includes residues in and around the highly conserved “WG” (TrpGly) sequence within the “Sep4” motif defined by Pan et al. (Pan et al., 2007). Although the β meander is not a canonical GTPase motif, it is near the pocket and includes Cdc10 Arg251 (in the “-4” position relative to the WG) which, as described above, contacts bound GDP in the crystal structure (Figure 4). In the crystal structure Cdc10 Asp182 is within 5 Å of the WG Trp of Cdc10, Trp255 (Figure 5C). Substituting Asp with Asn slightly repositioned Trp255, eliminating a contact with Cdc3 Tyr352 (in the “-2” position; Figure 5C). Thus, our approach is sensitive and, at least in this example, pinpoints evolutionarily relevant residues.

Some residues that contact nucleotide in the crystal structure (*e.g.*, Cdc3 Asp128) instead make interface contacts in the nucleotide-free AlphaFold predictions. In fact, in the wild-type prediction, Cdc3 Asp128 contacts Cdc10 Lys155, which replaces the canonical *trans* loop 1 His and which in the crystal structure contacts the γ phosphate of the GTP bound in Cdc3’s pocket (Figure 5D). The negatively charged Asp sidechain essentially substitutes for the negatively charged phosphate. While predicted interactions of this sort represent a caveat of our approach for septins that bind a phosphorylated nucleotide, for class I septins that do not bind a phosphorylated nucleotide, such predicted interactions may be authentic.

### Rapid G interface evolution in sporulation septins

Since *trans* loop 1 residues contact the partner’s nucleotide or residues thereabouts, we looked for evolutionary co-variation between *trans* loop 1 residues and presumptive changes in bound nucleotide. Like Cdc10, Spr3 has Lys instead of the more common *trans* loop 1 His (Figure 2). Spr28 has Arg (Figure 2). The partner of *S. pombe* Spn7, Spn6, has Phe (Figure 2), and this residue is Tyr in Spn6 from the closely related fission yeast species *S. octosporus* and *S. cryophilus*, and Cys in *S. japonicus* (not shown). Furthermore, a G4 Thr that also contacts the partner’s nucleotide in most septin crystal structures is Leu in Spr3, Gly in Spr28, and Ala in Spn7 (Figure 2). We interpret this unusual variability in otherwise highly conserved contacts as evidence of evolution of the interface to accommodate changes in the partner’s pocket.

**FIGURE 6:**
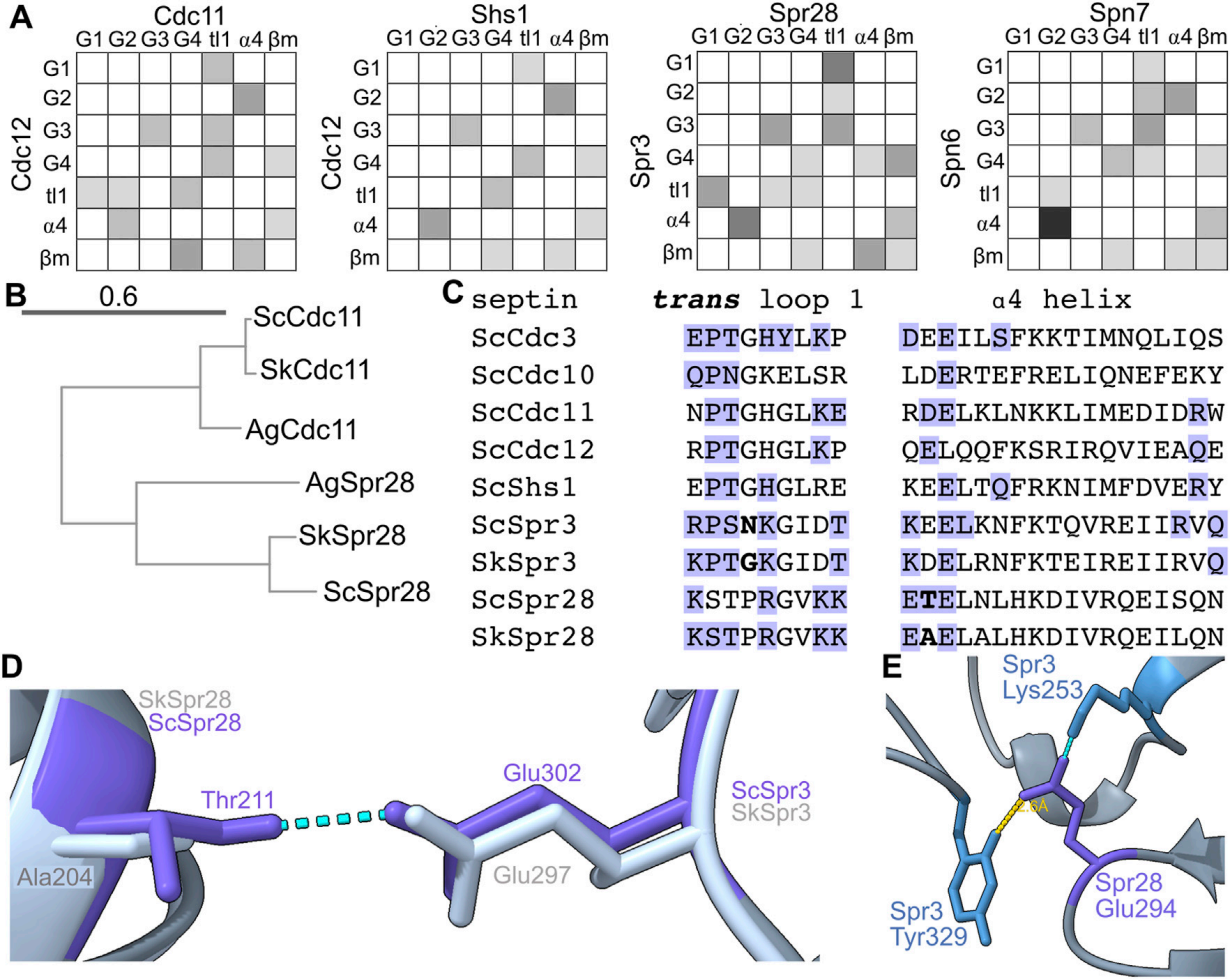
Evidence of rapid evolution of G interface contacts in sporulation septins. **(A)** As in Figure 5A but for the indicated heterodimers. **(B)** Phylogenetic tree showing evolutionary relationships between Cdc11 homologs in *S. cerevisiae* (“Sc”), *S. kudriavzevii* (“Sk”), and *Ashbya gossypii* (“Ag”). Accession numbers: EJT42412, NP_986841, P32458, Q04921, NP_985300.1, EJT41514.1. Scale bar unit is substitutions per site. **(C)** Protein sequence alignment for the *trans* loop 1 and ⍺4 helix for the indicated septins. “Sc,” *S. cerevisiae*; “Sk,” *S. kudriavzevii*. Blue text indicates G interface contacts either experimental (*Sc*Cdc3 and *Sc*Cdc10 from crystal structure) or predicted. Bold text highlights differences between *S. cerevisiae* and *S. kudriavzevii* discussed in the text. **(D)** The predicted structures of the Spr3–Spr28 heterodimer from *S. cerevisiae* and *S. kudriavzevii* were superimposed. Dashed blue lines indicate hydrogen bonds. **(E)** As in **(D)** but showing only the *S. cerevisiae* structure; the yellow line indicates distance.

Spn6 shows other signs of evolutionary “adaptation” to maintain the G dimer interface despite the absence of β and γ phosphates in the Spn7 pocket. The ⍺4 helix, which lies just C-terminal of the G4, is not usually considered a major site of G interface contacts; in the first septin crystal structure, only a single ⍺4 residue was identified as part of the interface (Sirajuddin et al., 2007). The situation for the Spn6–Spn7 prediction is very different: four ⍺4 residues in Spn6 make a total of six interface contacts, five with G2 residues in Spn7 (Figure 6A). If the Spn7 pocket is emptier than those of septin GTPases (lacking phosphates or nucleotide altogether) then perhaps alternative contacts evolved to maintain the interface with Spn6.

Within the genus *Saccharomyces* Spr28 evolved quickly relative to other septins: whereas Cdc11 is 95% identical between *S. cerevisiae* and *S. kudriavzevii*, Spr28 is only 80% identical between the two species. (Identity was calculated for full-length proteins.) Between *S. cerevisiae* and the non-*Saccharomyces* fungus *Ashbya gossypii*, Spr28 is 45% identical, compared to 81% for Cdc11. Figure 6B shows a phylogenetic tree for Spr28. Its G dimer partner, Spr3, is also less conserved (83% identical *S. cerevisiae* versus *S. kudriavzevii*, 48% identical *S. cerevisiae* versus *A. gossypii*). Most changes are conservative (retaining size/charge/hydrophobicity). Two non-conservative changes at the G interface stood out.

First, at a position in the *trans* loop 1 that is adjacent to the residue that often contacts the partner septin’s nucleotide and is Gly in *Sk*Spr3 and most other septins, *Sc*Spr3 instead has a residue with a long sidechain, Asn (Figure 6C). This difference is reminiscent of the variation in *trans* loop 1 residues we discussed above for Spn6. Second, an unusual ⍺4 Thr in *Sc*Spr28 that makes a predicted G interface contact with *Sc*Spr3 is replaced by Ala in *Sk*Spr28, and the Ala is not predicted to make any interface contact (Figures 6C, D).

The β meanders of sporulation septins also show particularly drastic differences from consensus at sites of interface contact. For example, at the “-1” relative to the WG, Spn7 has a very unusual Gln, Spr3 has Lys, and Spr28 has Glu (Figure 2). The “-2” position is also known to be important for G interface stability: a Thr in this position in human Septin-3 weakens the G interface with Septin-7, and substituting Thr to the more common Tyr restores a strong G dimer (Rosa et al., 2020). At this position Spn7 has a highly unusual Phe (Figure 2). In an AlphaFold prediction of the Spr3–Spr28 G interface, Spr28 Glu294 (“-1” residue) is within 3 Å of Spr3’s unusual Tyr329 (“+10” residue, Figure 6E) which may contribute to interface stability. Thus, the sporulation-specific septin G interfaces in yeasts experienced an unexpectedly high amount of variation, which may reflect “adaptation” to variable nucleotide occupancy in the pockets of these septins.

### Evidence that distinct G interface contacts co-vary with GTPase activity in nematode septins

**FIGURE 7:**
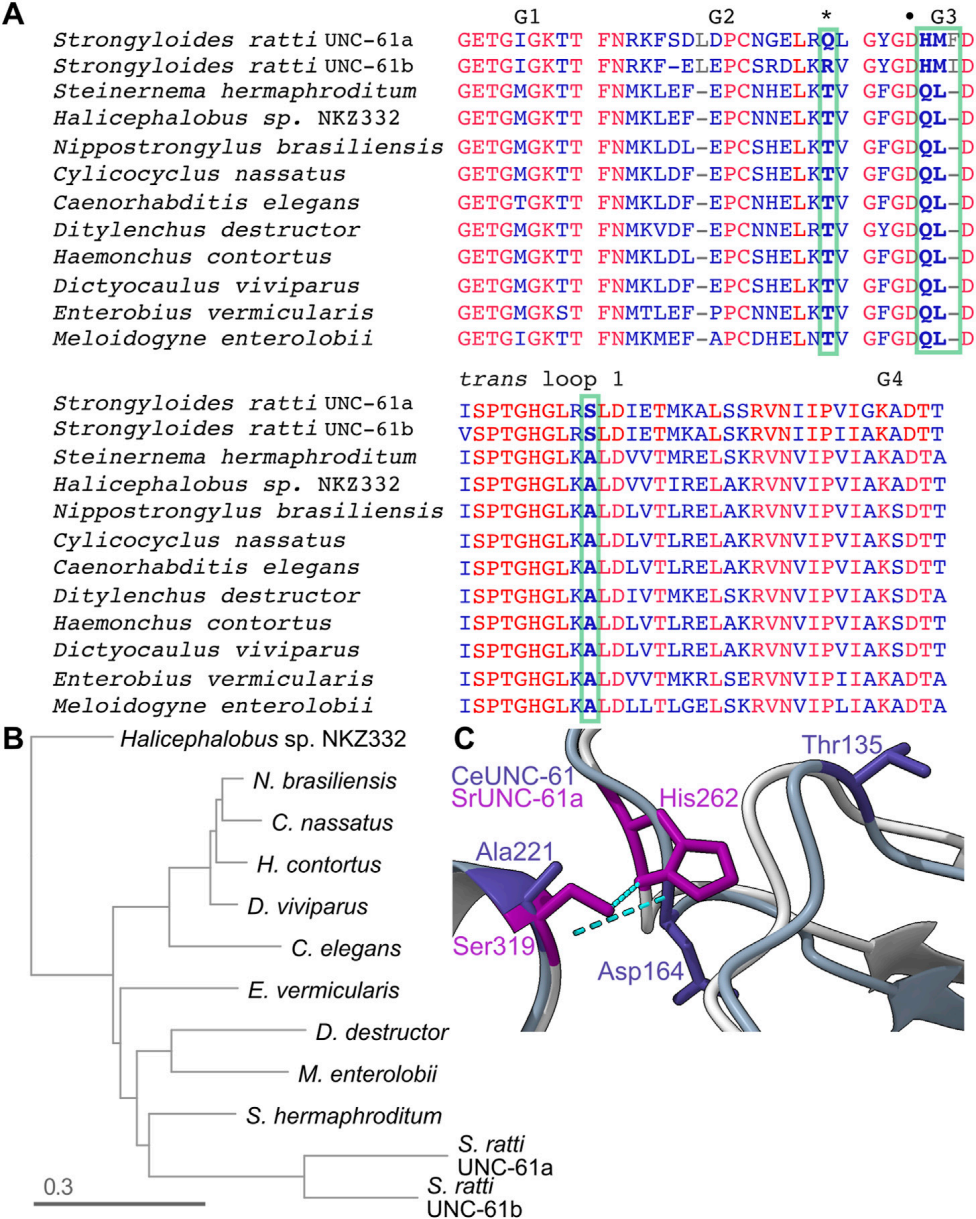
Rapid evolution in nematode septins following loss of GTPase activity. **(A)** Sequence alignment of specific regions of the UNC-61 homologs from the indicated nematode species. Red indicates identity. Bold text and green boxes indicate the position corresponding to the catalytic Thr (also marked by an asterisk) and residues that co-vary with the catalytic Thr and are at or near sites of predicted contact across the G homodimer interface. The bullet point indicates the G3 Asp that contacts the *trans* loop 1 Ala in **(C)**. Accession numbers: KAK0406599.1, KAE9549512.1, WKX94742.1, CAJ0608469.1, NP_506638.2, KAI1730409.1, XP_024506053.1, CDJ88949.1, KJH45839.1, VDD93301.1, CAD2194965.1, XP_024504518.1. **(B)** Phylogenetic tree for the UNC-61 homologs in **(A)**. **(C)** Superposition of predicted *Ce*UNC-61 homodimer (darker colors) with predicted *Sr*UNC-61a homodimer (lighter colors), showing the side chains of the indicated residues and hydrogen bonds as dashed cyan lines. *Ce*UNC-61 Thr135 is the presumptive catalytic Thr.

The existence of a class II septin pseudoGTPase (*S. mansoni* Septin-10) in the same phylogenetic group as *C. elegans* UNC-61 inspired us to look for additional pseudoGTPases in similar species. By examining other nematode septins, we found that the two UNC-61 homologs in the rat threadworm *Strongyloides ratti* (66% identical to each other, here referred to as “UNC-61a” and “UNC-61b”) both lack the catalytic Thr (Figure 7A). Thus during evolution of the group 1a septins the catalytic Thr (and, presumably, GTPase activity) was lost at least three independent times. UNC-61 forms a G homodimer in the *C. elegans* septin hetero-tetramer (John et al., 2007). *S. ratti* UNC-61a and UNC-61b diverged rapidly (Figure 7B), which may reflect both a relaxation of selection on residues involved in GTP hydrolysis or GDP binding, and rearrangement of G interface contacts to adapt to a different nucleotide in the pocket. In *Sr*UNC-61b, the catalytic Thr is replaced by Arg, as opposed to Gln in *Sr*UNC-61a (Figure 7A); for a pseudoGTPase any non-Thr residue at this position may suffice.

We expected *S. ratti* UNC-61a and UNC-61b to form either G homodimers or heterodimers, depending on if they are expressed in the same cells. Either way, a G dimer involving two GTP-bound septins would represent a scenario without experimental precedent and thus would presumably require changes to the G interface. To look for such changes, we compared the intermolecular hydrogen bonds within the AlphaFold predictions of *Ce*UNC-61 and *Sr*UNC-61a G homodimers. Notably, the five top-ranked *Sr*UNC-61a predictions were NC homodimers (not shown), so we used the sixth ranked prediction, which had equivalent confidence scores for the residues in question compared to the top-ranked *Ce*UNC-61 prediction (Supplementary Figure S3). Of the few differences, two were noteworthy: whereas in *Ce*UNC-61 the monomers’ G3 loops interacted via bonds between a Lys and a Leu, and a G3 Asp contacted a *trans* loop 1 Ala, in *Sr*UNC-61a the Ala is replaced by Ser and the contact with the G3 Asp was lost (Figure 7C). [We note that this G3 Asp is the equivalent of Asp210 in Cdc3 that, when mutated to Gly, stabilizes the Cdc10(D182N)–Cdc3(D210G) interface (Weems et al., 2014) (Figure 2)]. Instead, the *trans* loop 1 Ser of *S. ratti* UNC-61a contacted a G3 His (Figure 5D). The G3 in *S. ratti* UNC-61a has additional changes: the Leu is replaced by Met, and a Phe is inserted in the loop (Figure 7C). Among 11 nematode species that we examined, the presence of the His and the Leu-to-Met and Ala-to-Ser substitutions correlated perfectly with absence of the catalytic Thr (Figure 7A). Since in active septin GTPases the G3 changes conformation depending on the phosphorylation state of bound nucleotide, these observations are consistent with changes to the G interface to accommodate γ phosphate and the corresponding repositioning of the “switch” loops (G2 and G3).

### Summary and perspectives

While septins have not previously been considered among the pseudoGTPases (Stiegler and Boggon, 2020), direct experimental evidence has clearly established lack of GTPase activity but retention of GTP binding for multiple septins. The existence of class II septin pseudoGTPases is therefore unambiguous. The evidence we provide here supports Spn7 from *S. pombe* as being a class I pseudoGTPase (unable to bind GTP or GDP) though confirmation awaits direct experimental tests of nucleotide binding. Spr28 from *S. cerevisiae* remains more ambiguous, since in our predictions it bound well to GDP. Our analysis supported our prediction that Shs1 is a Class III septin pseudoGTPase, whereas to our surprise Cdc11 appeared to be able to bind both GTP and GDP and possibly hydrolyze GTP.

With regard to evolution of the G dimer interface, we found multiple examples of non-conservative substitutions in predicted contacts that correlated with predicted changes in the identity of the nucleotide bound in the pocket of at least one of the septins involved in the dimer. We note that, apart from the limitations inherent in relying on *in silico* predictions, our models implicitly assume a typical protein folding environment, which may be misleading. While we considered alterations in nucleotide availability during sporulation, for practical reasons we did not consider fundamental changes in the environment in which septins fold and interact with each other and with nucleotides. For example, a huge increase in the concentration of intracellular trehalose during yeast sporulation (Roth, 1970) likely stabilizes proteins and protects against aggregation (Singer and Lindquist, 1998; Jain and Roy, 2009). We previously demonstrated how other naturally occurring small molecules like guanidine and trimethylamine N-oxide can restore viability to septin-mutant yeast cells with single substitutions in canonical pocket residues (Johnson et al., 2020; Hassell et al., 2021). Temperature also has a tremendous impact on the functional consequences of changes to septin G interface contacts. Thus, some septin sequence variation during evolution may reflect adaptation to changes in the physicochemical environment that we do not yet understand.

Similarly, our sequence-based analysis cannot capture potential influence of post-translational modifications on pocket/interface residues. Examples include phosphorylation of Cdc10 Ser256 (a very unusual, *Saccharomyces*-specific residue in the “+1” position of the “WG”) (Versele and Thorner, 2004) and Ser198 of human Septin-12 (equivalent to Cdc10 Thr183 that contacts GTP bound by Cdc3) (Shen et al., 2017). Septin-interacting proteins may also alter the G interface in ways that our analysis cannot account for. Cytosolic chaperones are the only non-septin proteins known to directly contact the septin G interface (Hassell et al., 2022), yet the *Drosophila* Orc6 protein somehow stimulates the GTPase activity of purified septin hetero-oligomers despite binding the septin coiled-coil forming sequences far from the GTPase domain (Huijbregts et al., 2009). We previously noted (Weems et al., 2014) that the yeast septin Cdc12 has a C-terminal sequence homologous to a sequence in Ran that, when deleted, allosterically alters the G2 and G3 regions of Ran•GDP to mimic the GTP-bound state (Nilsson et al., 2002). Thus, interaction of non-septin proteins like Orc6 with septin regions distal from the GTPase domain may allosterically alter the G interface contacts of septin pseudoGTPases in a manner that our approaches cannot predict. Nonetheless, taking our new observations together with the extant literature [see (Abbey et al., 2019)], it is probably time to stop referring generally to septins as “GTPases.”

## Data Availability

The datasets presented in this study can be found in online repositories. The names of the repository/repositories and accession number(s) can be found below: https://doi.org/10.6084/m9.figshare.24158691.v1.
