# Supplementary material for: Evolutionary degeneration of septins into pseudoGTPases: impacts on a hetero-oligomeric assembly interface
Source: Front Cell Dev Biol. 2023 Nov 29;11:1296657. doi: 10.3389/fcell.2023.1296657 (PMC10731463; doi:10.3389/fcell.2023.1296657)
Supplement: Supplementary file 1 [file DataSheet1.docx]

Supplementary Material

# Supplementary Figures
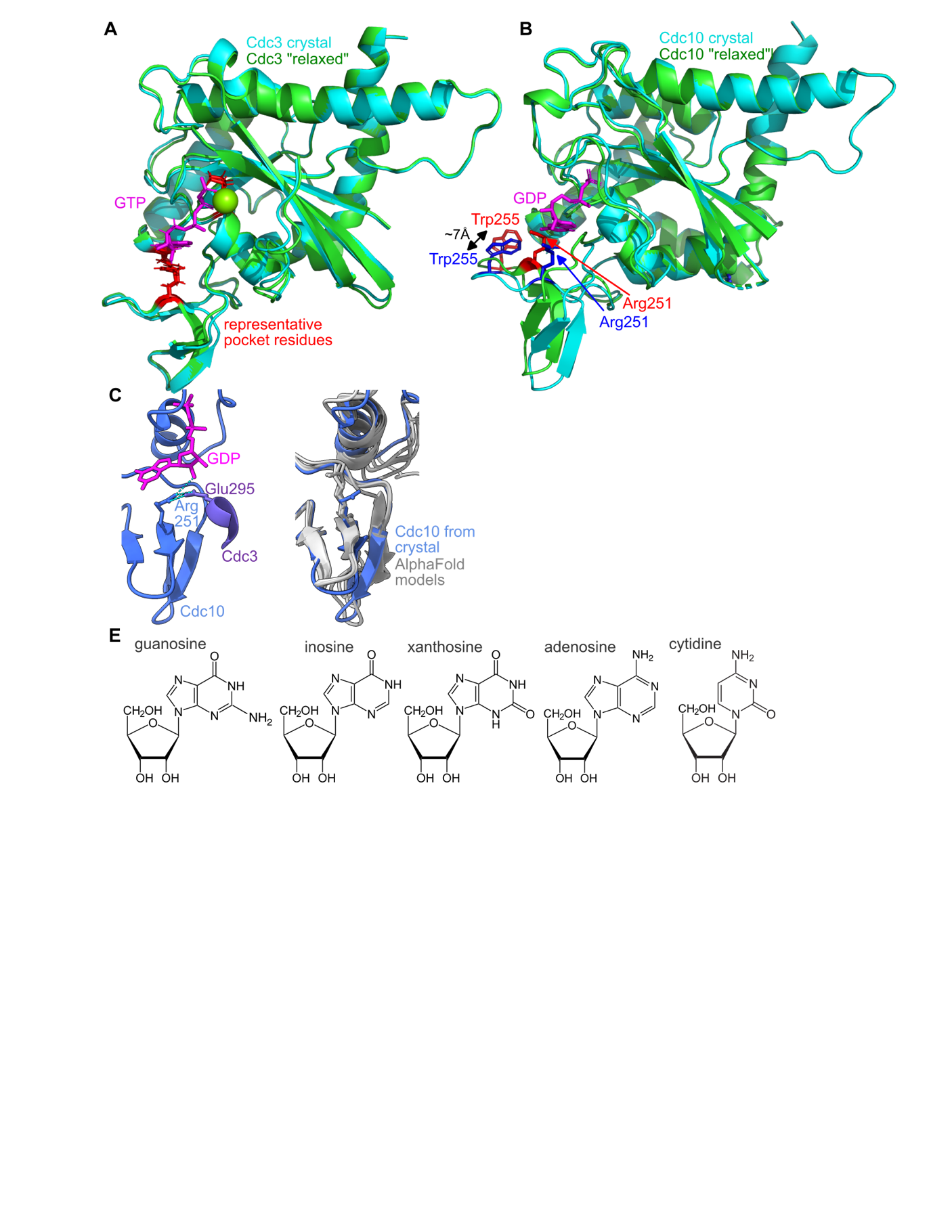


**Supplementary Figure 1. Collapse of a part of the pocket upon *in silico* energy minimization in the absence of nucleotide.** (A) Superposition of the “GTPase” domain of Cdc3 as it appears in the Cdc3–Cdc10 heterodimer crystal structure (PDB 8SGD, blue) (22) versus following *in silico* GTP removal and energy minimization (green). A few pocket residues are shown in stick form in red. Nucleotide is in magenta. (B) As in (A) but for Cdc10 following removal of GDP. Arg251 and Trp255 from the crystal structure or the relaxed structure are shown as blue or red sticks, respectively. (C) A part of the Cdc3–Cdc10 crystal structure showing the orientation of Cdc10 Arg251 and hydrogen bonds (dashed cyan lines) to bound GDP and Glu295 from Cdc3. (D) Similar pose as in (C) but for superposition of the AlphaFold predicted structures of Spn7, Spr28, Cdc11, and Shs1 with Cdc10 from the Cdc3–Cdc10 crystal structure, showing the side chains of Cdc10 Arg251 and the equivalent residues from the other septins. (E) Chemical structures of the nucleosides used for docking.

**
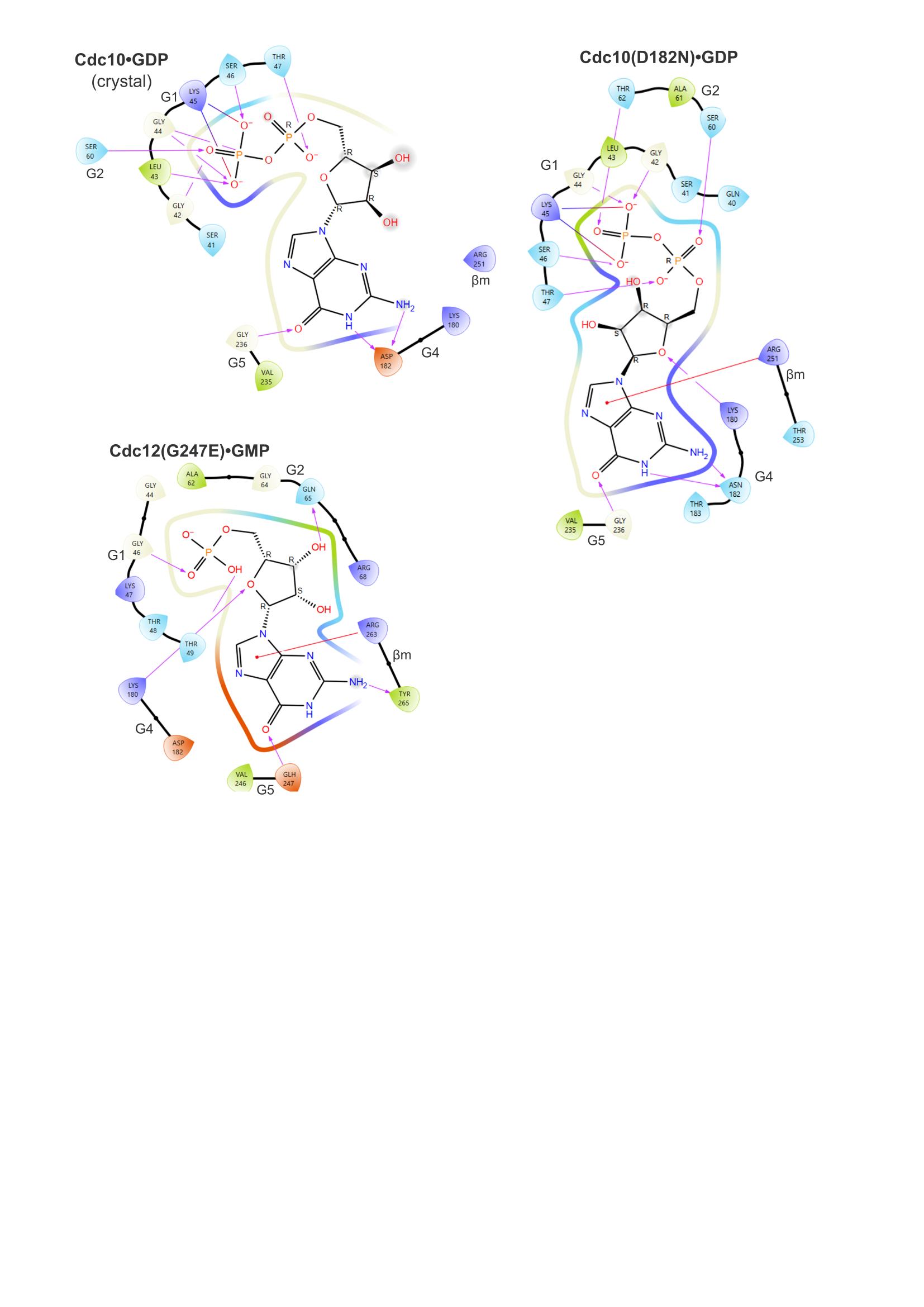
**

**Supplementary Figure 2. Predicted contacts between mutant septins and bound nucleotide according to *in silico* docking.** As in Figures 3 and 4 but for the indicated *S. cerevisiae* septins carrying single substitutions identified in temperature-sensitive mutant screens.


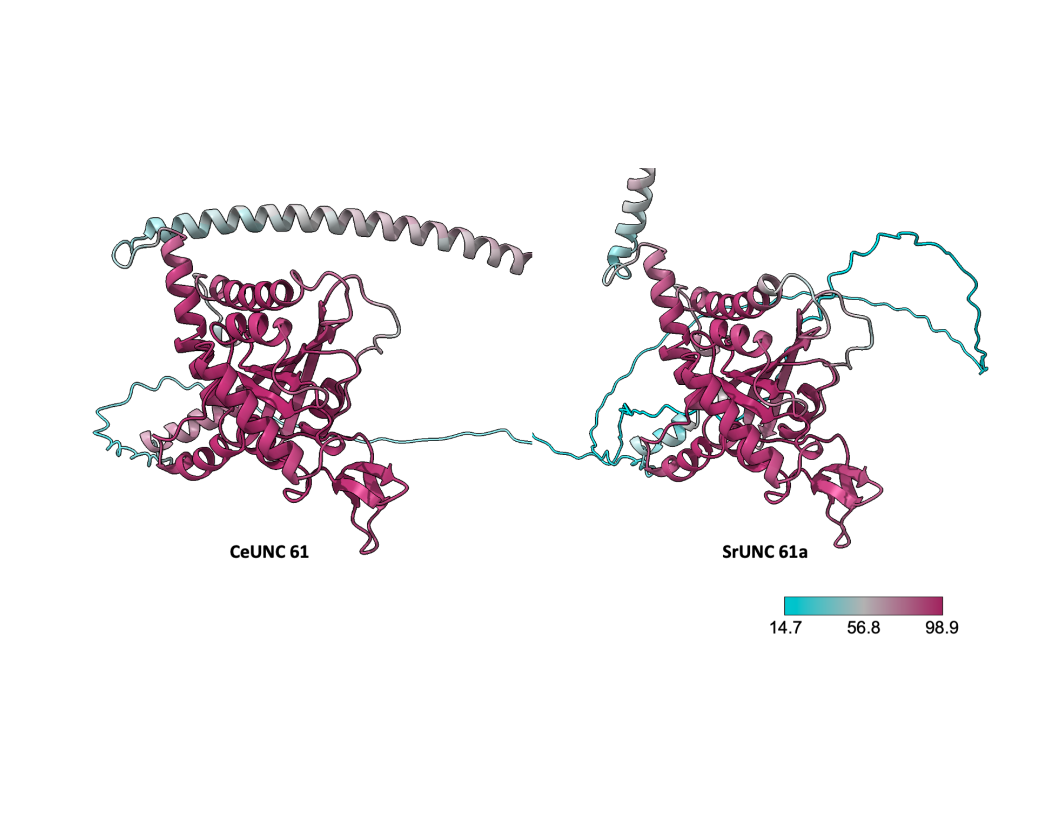


**Supplementary Figure 3. AlphaFold confidence scores overlaid on predicted septin structures.** For the top-ranked homodimer structure produced by AlphaFold Multimer for *C. elegans* UNC-61 and the 6^th^-ranked homodimer structure for *S. ratti* UNC-61a, one monomer of each is shown with each residue color-coded according to its AlphaFold confidence score for the homodimer prediction.
